# Chemical Structure of Stabilizing Layers of Negatively Charged Silver Nanoparticles as an Effector of Shifts in Soil Bacterial Microbiome under Short-Term Exposure

**DOI:** 10.3390/ijerph192114438

**Published:** 2022-11-04

**Authors:** Sebastian Wojciech Przemieniecki, Magdalena Oćwieja, Sławomir Ciesielski, Wiktor Halecki, Ewelina Matras, Anna Gorczyca

**Affiliations:** 1 Department of Entomology, Phytopathology and Molecular Diagnostics, University of Warmia and Mazury in Olsztyn, Prawochenskiego 17, PL-10721 Olsztyn, Poland; 2 Jerzy Haber Institute of Catalysis and Surface Chemistry Polish Academy of Sciences, Niezapominajek 8, PL-30239 Krakow, Poland; 3 Department of Environmental Biotechnology, University of Warmia and Mazury in Olsztyn, Słoneczna 45G, PL-10719 Olsztyn, Poland; 4 Institute of Nature Conservation, Polish Academy of Sciences, Mickiewicza 33, PL-31120 Kraków, Poland; 5 Department of Microbiology and Biomonitoring, University of Agriculture in Krakow, Mickiewicza 21, PL-31120 Krakow, Poland

**Keywords:** silver nanoparticles, surface properties, fungicide, soil bacteria, biochemical activity

## Abstract

In this work, we have assessed the exposure of soil bacteria from potato monoculture to three types of silver nanoparticles (AgNPs) as well as silver ions (Ag^+^ ions) delivered in the form of silver nitrate and a commercially available fungicide. The diversity of the soil microbial community, enzymatic activity, and carbon source utilization were evaluated. It was found that only the fungicide significantly limited the abundance and activity of soil bacteria. Silver ions significantly reduced bacterial metabolic activity. In turn, one type of AgNPs prepared with the use of tannic acid (TA) increased bacterial load and activity. There was found in all AgNPs treated soils (1) a greater proportion of all types of persistent bacteria, i.e., *Bacillus*, *Paenibacillus,* and *Clostridium*; (2) a visible decrease in the proportion of *Nocardioides*, *Arthrobacter,* and *Candidatus* Solibacter; (3) almost complete depletion of *Pseudomonas*; (4) increase in the number of low-frequency taxa and decrease in dominant taxa compared to the control soil. Despite the general trend of qualitative changes in the bacterial community, it was found that the differences in the chemical structure of the AgNP stabilizing layers had a significant impact on the specific metabolic activity resulting from qualitative changes in the microbiome.

## 1. Introduction

Bacteria are some of the most important living components of the soil. Bacteria abundance (about billion cells) and species diversity (min. 4000–7000 genomes) per gram of soil cause their great but also variable metabolic activities. Bacterial communities are heavily implicated in energy and nutrient exchanges within the soil by a key role in the biogeochemical cycles of the main and trace elements. The reaction of the bacteria at the moment to various natural or man-made disturbances could be answered on soil conditions [1]. It has been proven that bacterial community composition and activity correlate with soil health. It has been shown also that changes in the soil environment, largely brought about by anthropogenic activity, correlate more strongly with changes in bacterial community composition than with spatial factors [2]. In recent years, the development of Next-Generation DNA Sequencing (NGS) techniques in metagenome analysis has resulted in the creation of highly reliable methods for monitoring the environment, including the condition of soil modified by certain factors. This attractive avenue of research helps to assess quickly and efficiently bacterial communities’ data which are metrics of environmental health and soil production potential [3,4].

Metal nanoparticles (MNPs) are the most extensively studied type of nanomaterials due to their properties and, consequently their wide utility. Applications of MNPs can be found in everyday life and high technology. Producers of textiles, cosmetics, food packaging, paints, water repellent, antifog, antimicrobial surface coatings, disinfectants, fertilizers, pesticides, fuel additives, medical dressings, dental materials, vehicles for gene and drug delivery, polymers, high-tech optical devices, highly selective and sensitive sensors, and filters, as well as appliances used in water treatment and remediation processes use MNPs [5,6,7,8,9,10,11,12,13]. A special boom in implementations occurred at the beginning of the 20th century. Currently, the production and application of different MNPs are already leading to the release of thousands of tons of them into the environment. Every year, the most frequently applied MNPs (e.g., gold, silver, platinum) and metal oxide (Me_x_O_y_NPs) (Al_2_O_3_, CeO, Cu_2_O, Fe_2_O_3_, Fe_3_O_4_, ZnO, TiO_2_, SiO_2_) most often end up in the soil, either directly or via landfill in the form of sediments and waste. These nanomaterials are also released into the water and the atmosphere [14,15,16,17]. It is worth emphasizing that AgNPs are at the forefront of nanostructures produced and released into the environment and have a high biocidal potential against microorganisms, which can modify ecosystems [18,19,20].

In the field of agriculture, many studies have shown that MNPs significantly affect organisms in agroecosystems, including in a toxic way. MNPs can affect the plant and soil microbiome in both a harmful and beneficial way [9,21]. Their impact has often been observed in the form of disruption of the functioning of microorganisms at the cellular level. The exposure to diverse types of MeNPs is often manifested as an increase in the permeability of cell membranes, an overproduction of reactive oxygen species, disturbances in the enzymatic regulation of cells, and interruptions of replication of deoxyribonucleic acid [22]. However, many studies on the impact of the same MNPs on selected cells and organisms have differed significantly in their results, which were often contradictory depending on the group of researchers. This is due to the use of MNPs that differ in size, shape, surface, and electrokinetic proprieties, which had an influence on the results. At the same time, it was also observed that, depending on the organism used in the study, different results were obtained, i.e., in one species and even one strain/ecotype, MNPs of a given caused toxicity while in others, they caused stimulation [23,24,25,26].

In the case of the application of MNPs to the agricultural environment (i.e., fertilizers or pesticides), it is very important to thoroughly understand the mechanisms of bioactivity and the fate of MNPs. In this specific ecosystem, the impact of AgNPs, especially in the soil environment, is not yet fully understood. A great deal of research on soil microbiota has focused mainly on comparing various MNPs. However, there is no unambiguous information on the differences in the impact on the soil environment of MNPs of the same metal obtained by diverse methods of preparation.

Chemical, physical, and biological methods are used in the production of AgNPs. The method of production has implications in terms of specific properties of AgNPs and, as a consequence, different reactivity to cells [27]. Due to their high biological activity and well-documented biocidal properties, AgNPs are at the center of interest of toxicological research. Depending on the conditions of preparation, AgNPs exhibit diverse physicochemical properties, which have an effect on their differing impact on the environment. Studies that take into account many soil parameters, as well as the physicochemical properties of AgNPs (size, shape, surface charge, chemical structure of stabilizing layer, silver ion release profile), initially confirmed their different impact on the soil microbiome [28,29], which further emphasizes the need for more research to be conducted both on a larger scale and in greater detail.

In the chemical approach, diverse compounds playing a role of a metal precursors, reducing agents, and stabilizing agents, are involved in the reaction leading to the formation of AgNPs. In some instances, the reducing agent may also act as a stabilizer, or oxidized forms of the reducing agent fulfill the role of stabilizers of the AgNPs. The surface properties of formed AgNPs are related to the type of reducing agent/stabilizer used in the reaction [30,31,32,33].

Due to the presence of stabilizers, AgNPs take on a positive, neutral, or negative electrokinetic charge. The charge of AgNPs has an impact on interaction with biological membranes. It has been shown that changing the surface charge of AgNPs leads to a significant fluctuation in their activity with regard to cells. Because the surface of bacteria usually has a negative charge, positively charged AgNPs are strongly attracted to the surface of bacteria, resulting in an increased anti-bacterial activity. According to certain data from the literature, neutral or negatively charged AgNPs have a significantly reduced anti-bacterial action. However, an increase in the concentration of AgNPs allows the electrostatic repulsion to be weakened by a method of saturation of the surface of the bacteria [34,35]. It has also been shown that the surface properties of AgNPs play a decisive role in the adsorption of biomolecules (mainly proteins) onto the surface of NPs, forming a protein corona at the biointerface, which may also have a decisive impact on their toxicity in interaction with the cell [36,37].

There is a lack of systematic data in the literature showing whether the biological activity of identically charged AgNPs may differ due to the presence of chemically different stabilizers adsorbed onto their surface. Such studies may reveal the details of certain dependencies in relation to the release into the environment of AgNPs produced by chemical reduction.

Taking into consideration the significance of the soil bacteria and their importance in soil fertility and ecosystem services, it was planned to conduct studies concerning the impact of spherical and negatively charged AgNPs of comparable size distribution obtained in a chemical reduction method on the bacterial component of the soil microbiome. The objectives of the study were (1) to investigate that the reducing and stabilizing substances used in the production of negatively charged AgNPs, as well as modification of their surface coating, have a significant impact in terms of changes in the structure and biochemical activity of the treated soil bacteria; (2) to determine the impact of AgNPs on the bacterial microbiome with respect to Ag^+^ ions and a commercial fungicide. The research hypotheses were: AgNPs change the microbiological state of the soil environment; that the degree of influence of AgNPs depends on the reducing and stabilizing substances used and depends on the surface coating; that AgNPs interact with soil bacterial microbiota and its potential biochemical activity in a way that is different to Ag^+^ ions and commercially available fungicide. 

## 2. Materials and Methods

### 2.1. Materials

Each reagent applied for the preparation of AgNPs was received from Avantor Performance Materials Poland S.A. and was used without any additional purification. Ultrapure water applied for the preparation of AgNP suspensions was produced by the Milli-Q Elix&Simplicity 185 purification system (Millipore SAS, Molsheim, France).

Commercially available fungicide contains two active substances: mancozeb (ethylene bisdithiocarbamate, C_4_H_8_N_2_S_4_) 64% (640 g·kg^−1^) and metalaxyl-M (*N*-(2,6-dimethylphenyl)-*N*-(methoxyacetyl)-D-alanine methyl ester, C_15_H_21_NO_4_) 3.8% (38.8 g·kg^−1^) was used as pesticide variant.

The soil used in the experiments was obtained from potato monoculture crops located at the Production and Experimental Plant in Balcyny (53°35′51.0″ N 19°50′46.4″ E, Warmia and Mazury Voivodeship, Poland). The soil was sieved through a 2 mm sieve and analyzed according to applicable, commonly used standards. It was established that the pH of the soil was equal to 5.1. The content of P_2_O_5_, K_2_O, and Mg was equal to 16.3, 14.5, and 3.7 mg/100 g^−1^ of fresh soil mass, respectively, whereas the ratio of the mass of carbon to the mass of nitrogen was 8.36.

### 2.2. AgNPs Synthesis and Characteristics

Three types of AgNPs were obtained by a chemical reduction method of Ag^+^ delivered in the form of silver nitrate by selected reducing agents. The AgNPs were obtained in the form of aqueous suspensions according to the protocols described in the literature previously [38]. For the sake of convenience, the following abbreviations were introduced for the AgNPs in accordance with the names of reactants used in synthesis. AgNPs-TA were prepared with the use of tannic acid (TA) under alkaline conditions adjusted by the addition of ammonia solution. AgNPs-SBTC were obtained by the reduction in Ag^+^ ions by sodium borohydride (SB) in the presence of trisodium citrate (TC), playing the role of a stabilizing agent. AgNPs-SHSH were synthesized by the reduction in Ag^+^ ions by sodium hypophosphite (SH) in the presence of sodium hexametaphosphate (SH) [39].

After the synthesis, the AgNP suspensions were purified. For this purpose, the suspensions were washed with ultrapure water with the use of an Amicon filtration cell (Millipore, model 8400, Burlington, MA, USA) equipped with a PLHK07610 regenerated cellulose (for AgNPs-SBTC and AgNPs-TA) or a PBHK07610 polyethersulfone (for AgNPs-SHSH) membrane. The process was carried out until the conductivity of the dispersive medium attained value of 15 μS cm^−1^.

A CPC-505 pH-meter/conductometer equipped with an ERH-12-6 combined electrode (Elmetron) and an EC-60 conductometric sensor was used to determine the pH and conductivity of the stock AgNP suspension.

The mass concentration of AgNPs dispersed in the purified suspensions was determined based on measurements of the density of the suspensions and their dispersive media (the effluent obtained in the purification procedure) [40]. The density of the stock silver suspension (*ρ_s_*) and the effluent solution (*ρ_e_*) was measured using a DMA 5000 M densitometer (Anton Paar). The mass fraction (*w*) of AgNPs in the stock suspension was then determined from the relationship (where (*ρ_p_*) is a density of silver equal to 10.49 g cm^−3^):

(1) $$w=\frac{\rho_{p}\left(\rho_{s}-\rho_{e}\right)}{\rho_{s}\left(\rho_{p}-\rho_{e}\right)}$$

The measurements were conducted in triplicate.

The suspensions of AgNPs were investigated in the UV-vis range (200–700 nm) using a UV-2600 spectrometer (Shimadzu, Kyoto, Japan). The extinction spectra of suspensions of controlled pH and ionic strength were recorded with a 0.5-cm path-length quartz cell.

The morphology and size distribution of AgNPs were assessed based on micrographs obtained using a JEOL JSM-7500F scanning electron microscope (JEOL, Tokyo, Japan, working in transmission mode. MultiScan Base software v 18.03 (Computer Scanning System, Warszawa, Poland) was used for the analysis of the recorded micrographs.

The stability (changes in the diffusion coefficients and hydrodynamic diameters) and electrokinetic properties (electrophoretic mobility, zeta potential) of AgNPs were investigated using the Zetasizer Nano ZS apparatus (Malvern Panalytical Ltd., Malvern, UK) [40].

### 2.3. Soil Treatment with Tested Substances

The soil was treated with the AgNP suspensions and silver nitrate solution in a way that made it possible to obtain a final concentration of active substances (silver) equal to 10 mg kg^−1^ of soil. For this purpose, an amount of soil of 400 g per beaker was mixed with 40 mL of the mixtures of active substances with a concentration of silver equal to 100 mg L^−1^. Then, the samples were topped up with ultrapure water to obtain the proper concentration. Afterward, the soil was mixed thoroughly, and the humidity was regulated so that it amounted to 60% of the maximum water capacity. The incubation lasted 7 days in a climatic chamber according to the methodology presented by Gorczyca et al. [41]. The established protocol was also applied for the preparation of soil treated with a fungicide with the active substances metalaxyl (3.8%) and mancozeb (64%). The fungicide used is recommended for control of early blight caused by *Alternaria solani* and *A. alternata* and late blight caused by *Phytophthora infestans* in potato crops. Blights are a major cause of disease in potatoes. Fungicides for the control of potato blights are commonly used in a preventative manner, optionally in conjunction with disease forecasting. In susceptible varieties, sometimes fungicide applications may be needed weekly. This determines the high level of release into the environment and the possibility of modifying the soil microbiome, especially in potato monocultures.

The control was the untreated soil with maintained moisture. Each variant was repeated four times.

### 2.4. Extraction of Genomic DNA

The isolation of genomic DNA from 250 mg of each sample of the soil was conducted using the QIAampPowerFecal DNA Kit (Qiagen, Hilden, Germany). The DNA concentration in each sample was determined by fluorometric quantitation using a Quantus™ Fluorometer (Promega, Madison, WI, USA). The genetic material obtained from four independent measurements was used in further studies.

### 2.5. Bacterial Load

The concentration of bacterial genomic DNA was determined by the TaqMan assay using the Maxima Probe Real-time PCR Master Mix 2X (Thermo Fischer Scientific, Waltham, MA, USA). For this purpose, 2 µL of extracted DNA, 500 nM of primers (BAC338F 5‘-ACTCCTACGGGAGGC-3′ and BAC805R 5′-GACTACCAGGGTATCTAATC C-3′) and 200 nM of the probe BAC516F 5′FAM-TGCCAGCAGCC5GCGGTAATA-TAMRA3′ were mixed together to obtain a final reaction volume equal to 20 µL. The reaction profile was as follows: initial denaturation at 95°C for 10 min, followed by 45 cycles of denaturation at 95°C for 15 s, and annealing/elongation at 60°C for 60 s. Each sample was analyzed in duplicate. The TOPO^®^ TA Cloning^®^ Kit (Thermo Fisher Scientific, Waltham, MA, USA), with 16S rRNA from *Bacillus subtilis* A9, was used as a standard genomic plasmid.

### 2.6. 16S rRNA Amplicon Sequencing and Bioinformatics Analysis

The bacterial communities colonizing each sample of soil were determined by sequencing the V3-V4 region of the 16S rRNA gene. The 16S rRNA gene fragment was amplified with the PCR primers recommended for the Illumina technique based on the manufacturer’s protocol (Illumina, Inc., San Diego, CA, USA). The primers were developed by adding Illumina adapter overhang nucleotide sequences to the PCR primers specified by Klindworth et al. [42]. Amplicons were indexed using the Nextera^®^ XT Index Kit according to the manufacturer’s instructions. DNA was sequenced in Illumina MiSeq in 2 × 250 paired endmodes. The sequencing results were saved in FASTQ files and uploaded to the MetaGenome Rapid Annotation Subsystems Technology (MG-RAST) server for analysis [43]. Each file underwent quality control (QC) which included quality filtering (removing sequences with ≥5 ambiguous base pairs) and length filtering (removing sequences with a length ≥2 standard deviations from the mean). Illumina metagenomic datasets are available at MG-RAST under the following accession numbers: 4789663.3 (Fungicide_SP), 4789664.3 (Control_SP), 4789665.3 (Silver_nitrate), 4789666.3 (AgNP_TA), 4789667.3 (AgNP_SHSH), 4789668.3 (AgNP_SBTC).

### 2.7. Biochemical Properties of Microbiota

The enzymatic activity and carbon source utilization by the microbiota were determined using the API ZYM enzymatic test and the API 20 NE test, respectively. For this purpose, 100 mg of each sample of soil was homogenized in 10 oscillations/s^−1^ for 5 min. Then, the homogenates were centrifuged at 500× *g* to remove the solid particles. The supernatants obtained were preincubated at 25°C in peptone water with 1% TSA (Triptic Soil Broth, MERCK Millipore, Burlington, VT, USA) and 1% of AUX medium (bioMérieux, Marcy-l’Étoile, France) to enhance the biochemical activity of the microbiome. Suspensions cultured overnight were used in the API analysis. A 2-day incubation at a temperature analogous to the one which prevailed during soil incubation was applied to determine the biochemical activity of the microbiota.

### 2.8. Data Analysis

Taxonomic differences between metagenomes were analyzed using Statistical Analysis of Metagenomic Profiles (STAMP v. 2.1.3) [44]. The significance of the relative proportion difference in the taxonomic distribution of samples was performed using the two-sided Fisher′s exact test with the Newcombe-Wilson confidence interval method. Results with *q* < 0.05 were considered significant, and the unclassified reads were removed from analyses. The biological relevance of the statistic taxa was determined by applying a difference between the proportions of at least 1% and a twofold ratio between the proportions.

The counts and species composition of microorganisms were analyzed. The species diversity of the analyzed microorganisms was determined with the use of the Shannon diversity index (H’), Pielou’s evenness index (J’), and Simpson’s dominance index (λ). The relationships between the administered substrate, the microorganisms identified in soil samples, and their enzymatic activity were determined by Principal Component Analysis (PCA) with Pearson’s correlation, Agglomerative Hierarchical Clustering (AHC) using Bray-Curtis distance with Wards agglomeration method and Principal Coordinate Analysis (PCoA) calculated using Euclidean distance dissimilarities and the UPGMA method. The heat map was generated using the average agglomeration method.

The differences between mean values were determined by one-way analysis of variance (ANOVA) at a significance level of 0.05. Homogeneous groups were identified with the use of Tukey’s test. The Shapiro-Wilk test and the Levene test were performed to determine the normality of the distribution and the homogeneity of variance, respectively. The results were processed statistically and interpreted graphically in XLSTAT (Addinsoft, Paris, France) and Statistica v. 13.1 (Statsoft, Krakow, Poland).

## 3. Results

### 3.1. Physicochemical Properties of AgNPs and Their Suspensions

The three types of AgNPs were obtained in the form of aqueous suspensions. A chemical reduction in Ag^+^ions, delivered as silver nitrate, was applied for the preparation of AgNPs. For this reason, the biological activity of silver nitrate as an AgNP precursor was also assessed. The basic physicochemical properties of the solutions and suspensions of biologically active substances used in these studies are shown in Table 1.

The pH of the purified and diluted suspensions used in further experiments ranged from 5.8 (AgNPs-TA) to 6.3 (AgNPs-SHSH, AgNPs-SBTC). Taking into account the negligible differences between the measured values, the pH of the suspensions was not regulated.

**Figure 1:**
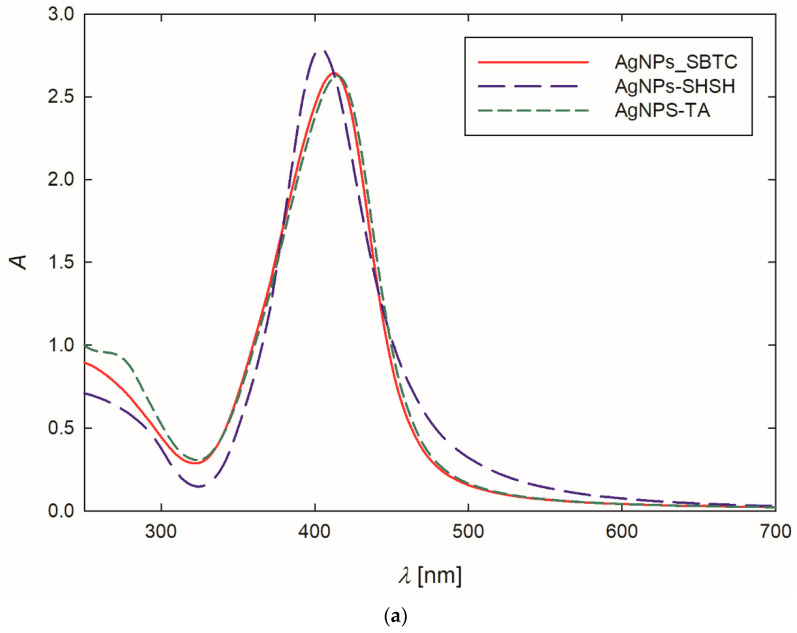

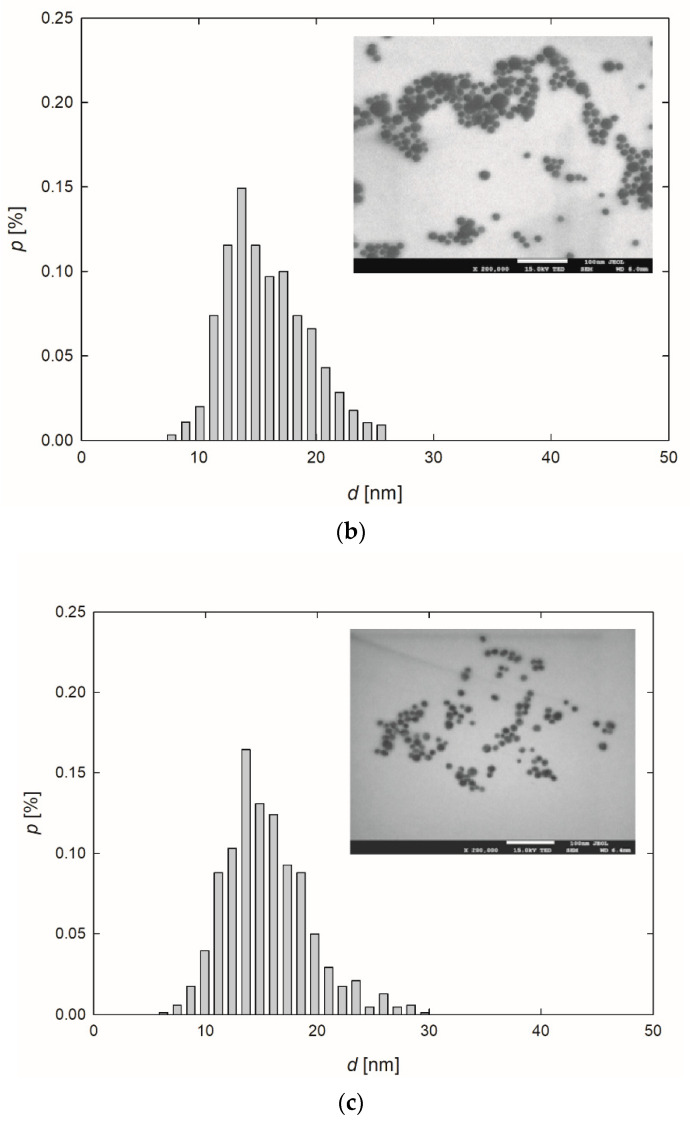

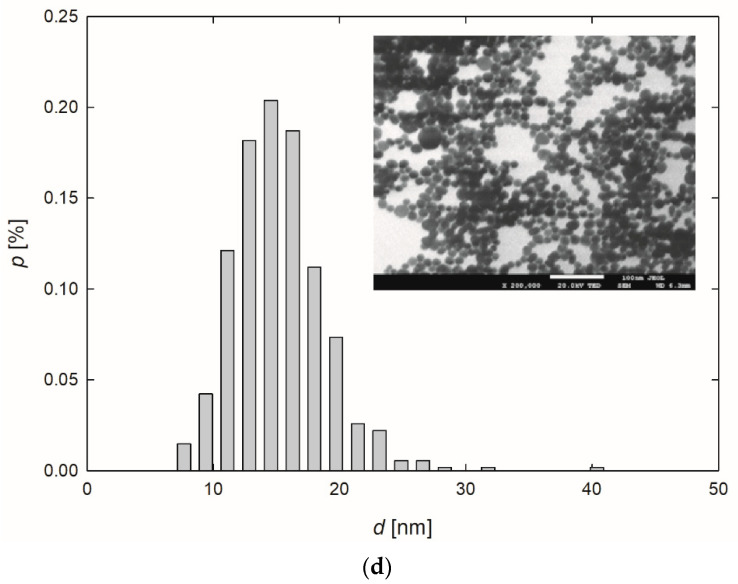
Extinction spectra (**a**) and the size distribution of AgNPs-TA (**b**), AgNPs-SBTC (**c**) and AgNPs-SHSH (**d**) with TEM micrographs (inserts).

At the outset, the formation of AgNPs was confirmed by recording the extinction spectra of the purified suspension. The stock AgNPs suspensions exhibited an intense yellow color. This observation was confirmed by the extinction spectra of the suspensions, where the maximum absorption bands, resulting from the occurrence of surface plasmon resonance [45], appeared at a wavelength of 404–414 nm (Table 1, Figure 1a). It is worth mentioning that the appearance of the absorption band below 400 nm indicates that relatively small AgNPs were obtained during the synthesis [46,47]. Analyzing Figure 1a, one can see that the bands are narrow and symmetric, exhibiting no shoulder in the region of higher values, which suggests that the AgNPs dispersed in the suspensions were fairly monodisperse, and they did not form larger aggregates [48,49].

Based on the TEM micrographs recorded, it was established that, independently of the preparation procedure, each type of AgNPs exhibited a nearly spherical shape (Figure 1b–d). The average size of the AgNPs-TA, AgNPs-SBTC, and AgNPs-SHSH was equal to 16 ± 4 nm, 15 ± 3 nm, and 14 ± 2 nm, respectively (Table 1).

The diffusion coefficients of AgNPs were determined in the suspensions of a concentration equal to 100 mg L^−1^ and at a temperature of 25 °C. The measurements were conducted immediately after the purification procedure and over the next 30 days. No significant changes in the values of diffusions coefficients were observed, which indicated that the AgNPs were stable under the conditions investigated. Knowing that the AgNPs exhibited a spherical shape and based on the values of diffusion coefficients obtained using the DLS technique, the hydrodynamic diameters were determined using the Stokes-Einstein relationship [40]. The results of the studies are presented in Table 1. Analyzing this data, one can see that the diameters of AgNPs determined using the microscopic technique and the DLS measurements remain in good agreement. This suggests that the thickness of stabilizing layers of NPs was negligible in comparison to the metal core.

The electrophoretic mobility measurements revealed that the AgNPs were negatively charged (Table 1). The measurements were carried out under the same conditions and for the same time periods as the DLS analysis. Based on the values of electrophoretic mobility and knowing the sizes of NPs, their zeta potential was calculated using Henry’s equation [40]. It is worth mentioning that the zeta potential values provide valuable information about the interactions of particles in colloidal systems, determining their stability and possible effects during the storage time. It was established that the zeta potential of the AgNPs-TA, AgNPs-SBTC, and AgNPs-SHSH was equal to −77 ± 4 mV,−75 ± 2 mV, and −63 ± 1 mV, respectively. One can notice that these values were significantly lower than the zeta potential of these AgNPs determined at an ionic strength of 10^−3^ M, which was described previously [38]. The time-dependent measurements did not indicate any significant variations in the zeta potential values, which suggests that the AgNPs were highly stable. 

Overall, three types of AgNPs characterized by a spherical shape, comparable size distribution, and negative surface charge, but diverse chemistry of stabilizing layers, were prepared. 

### 3.2. Effect of Soil Treatment of Bacteria

#### 3.2.1. Bacterial Load

**Figure 2:**
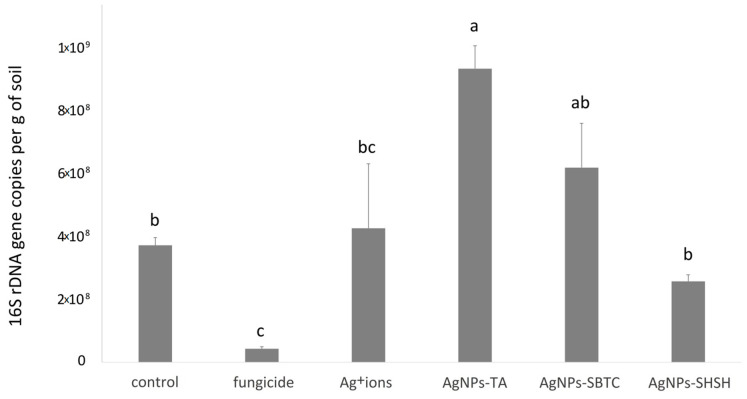
Average bacterial load in soil for experimental treatments after 7 days incubation. The values with the same letter are not significantly different (*p* =0.05).

It was found that all the tested substances modified the load of bacteria in the soil (Figure 2). Relative to the control variant, containing 3.9 × 10^8^ gene copies, the content of 16S rRNA (bacterial load) gene copies in soil changed significantly in two treatment cases. The highest number of gene copies was recorded (average 9.4 × 10^8^) in the variant treated with AgNPs-TA. In contrast, the variant treated with commercial fungicide was characterized by a distinct decrease to the level of 4.3 × 10^7^. In the other variants, there were no such significant differences in comparison to the control (Figure 2).

#### 3.2.2. Bacterial Community Structures

**Figure 3:**
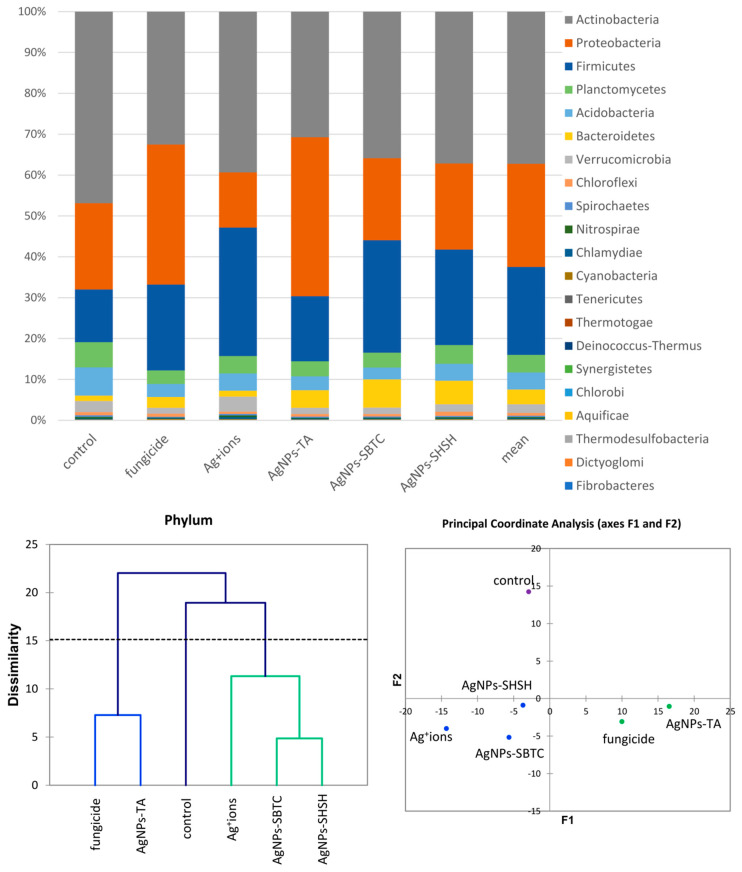
The structure of bacterial communities in treated soils on the level of phylum (**top**), Genetic dissimilarity between phyla represented by Agglomerative Hierarchical Clustering analysis (Bray-Curtis method) for the soils examined in the experiment (**bottom left**), and the relationship between the most abundant phyla depending on the treatments according to PCoA (**bottom right**).

An introductory analysis of microbiota diversity was performed at the phylum level. Based on the results of an analysis of frequency (Figure 3), it was observed that the most numerous phyla of microorganisms were Actinobacteria (average 37.2%), which were the most numerous in the control (46.9%) and the least numerous after fungicide and AgNPs-TA treatment (32%). The next most dominant phylum was Proteobacteria (average 25.3%), which were the most abundant in the AgNPs-TA variant (38.9%) and the least numerous after soil treatment with Ag^+^ ions (13.5%). The third most dominant phylum was Firmicutes (average 21.5%)—which were the most numerous in the Ag^+^ ions-treated variant (31.4%) and the least numerous in the control (12.9%). Of the other phyla, Bacteroidetes and Verrucomicrobia, although they were not dominant, were characterized by clear differences in frequency depending on the variant. The application of the AHC and PCoA methods allowed microbiomes that were different from each other to be put into groups. Of the three groups obtained, there was no difference in the soil bacterial community for the fungicide and AgNPs-TA-treated variants, nor in that of the group of the Ag^+^ ions, AgNPs-SBTC, and AgNPs-SHSH-treated variants. The microbiome of the control soil was most different from each of the treated soils (Figure 3).

**Figure 4:**
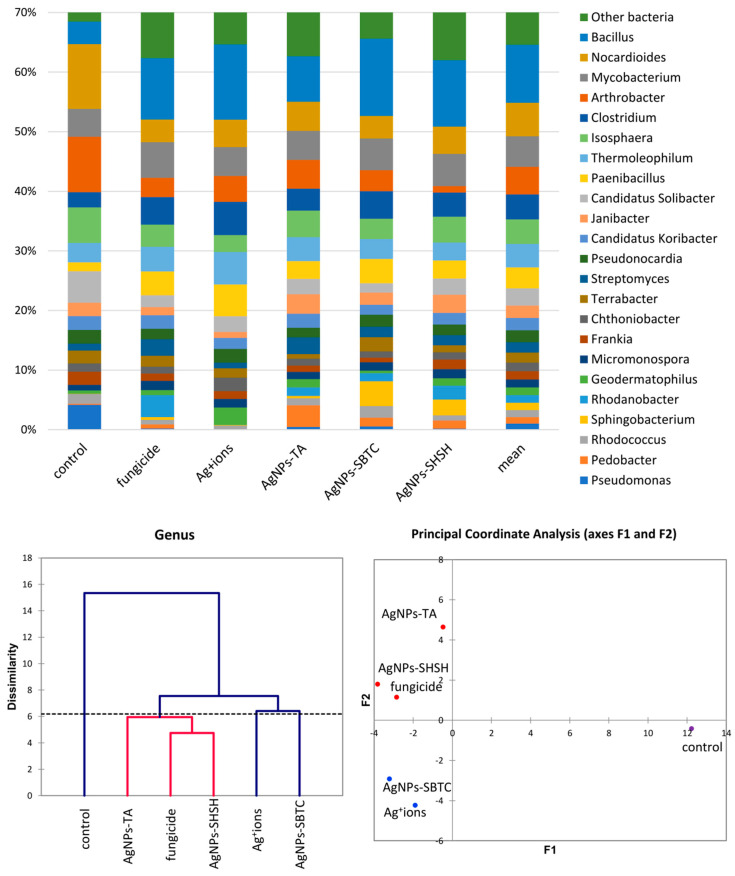
The structure of bacterial communities in treated soils on the level of genus (**top**), Genetic dissimilarity between genera represented by Agglomerative Hierarchical Clustering analysis (Bray-Curtis method) for the soils examined in the experiment (**bottom left**), and the relationship between the most abundant genera depending on the treatments according to PCoA (**bottom right**).

A more detailed analysis of the structure of the bacterial community was performed at the genera level. The frequency of *Bacillus* spp. clearly changed from 3.8% in the control to over 7.6% in the AgNP-TA-treated variant and over 10% in the other variants. In total, this genus was the only clearly dominant or subdominant genus occurring in treated soils. The share of *Clostridium* spp. also increased in each of the treated soils, although again, this was the least visible in the case of AgNPs-TA. *Paenibacillus* spp. apparently increased in the Ag^+^ ions-treated soil. The genera displaced under the treatments tested turned out to be *Nocardioides* spp. (inversely proportional to *Bacillus* spp.), *Arthrobacter* spp. (almost complete displaced in the AgNPs-SHSH variant), *Candidatus* Solibacter and, almost completely, *Pseudomonas* spp., a type that was highly abundant in the control (about 4.5%). Nevertheless, genera of bacteria unique to specific substances were observed. After the application of fungicide and AgNPs-SHSH, the frequency of the *Rhodanobacter* increased (from <0.1% in control to 3.7% and 2.3%, respectively). The Ag^+^ ions soil treatment resulted in an increase in the abundance of *Geodermatophilus* to 3% (0.6% ). *Pedobacter* spp. increased to 3.6% (0.2% in the control) in the AgNPs-TA variant. Lastly, the frequency of *Sphingobacterium* was <0.1% in the control soil, and this increased to 4.1% after AgNPs-SBTC treatment and to 2.6% after AgNPs-SHSH treatment (Figure 4).

The results of the analysis of differences at the level of the genus by the AHC (Bray and Curtis) and PCoA (Euclideas/UPGMA) methods (Figure 4) are similar regardless of the method used. Four groups differing from each other were distinguished. The fungicide, AgNPs-TA, and AgNPs-SHSH variants formed a common group with a low level of difference. In contrast, each of the other variants, namely the control, the Ag^+^ ions- and AgNPs-SBTC-treated variants, were different and created separate individual clades. Nevertheless, the results of PCoA indicate that AgNPs-SBTC and Ag^+^ ions are similar and fall on the same side of both coordination axes (coordination values for Ag^+^ ions F1 −1.90, F2 −4.23, and for AgNPs-SBTC F1 −3.21, F2 −2.92, with the control F1 12.24, F2 −0.42). The large dissimilarity of AgNPs-TA and the proximity to the coordination axis F2 indicate unique but insignificant differences in the frequency of several types (mainly *Bacillus* spp.), which can be observed on the graph. The very clear distinctness of the control was confirmed in each of the statistical analyses. The results of the proportion analysis confirmed the results of the metagenomic analysis statistically at the genus level (Figure S1). Spore-forming and some actinomycetes bacteria were the most resistant to the addition of the tested substances. The destructive effect of fungicide (opposite to the effect AgNPs-SBTC) on *Pseudomonas* and *Nocardioides* and a significant increase in the abundance of *Rhodanobacter* were confirmed (Figure 4).

**Figure 5:**
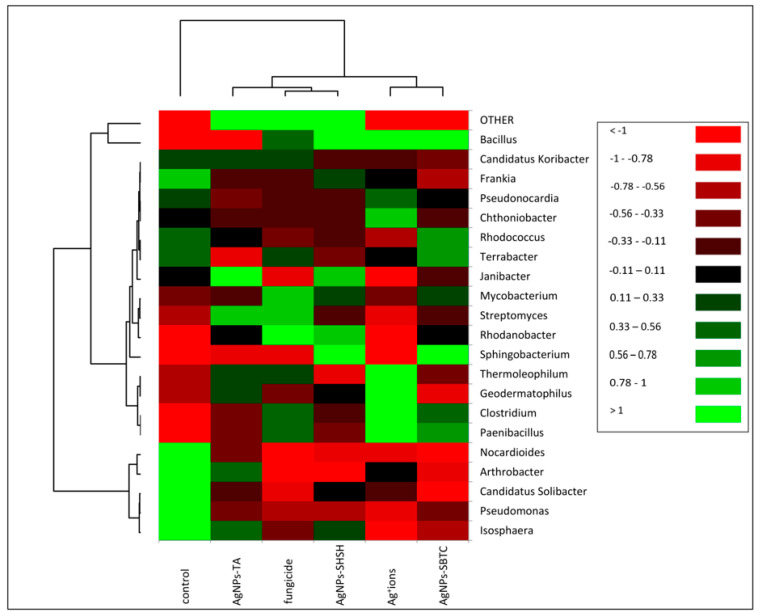
Heat map showing shifts in soil bacteria structure depending on the treatment.

The heat map allowed differences in the frequency of individual genera of bacteria to be determined in the examined soil (Figure 5). The upper dendrogram consists of three clades. However, in terms of the frequency of types, the control showed a clear separation (similar to the one presented in the PCoA) relative to the objects subjected to treatment. On the dendrogram determining the similarity between the frequencies of individual genera (left dendrogram), the formation of three main groups can be observed. The most different clade (bottom) contains fourgenera (i.e., *Arthrobacter*, *Candidatus* Solibacter, *Pseudomonas,* and *Isosphaera*) and shows objects where the relative frequency has decreased after the application of the tested agent. The second, largest clade consisting of 15 genera, was divided into two subgroups. The first subgroup consisted of four genera: *Thermoleophilum*, *Geodermatophilus*, *Clostridium,* and *Paenibacillus,* and showed a higher frequency in the Ag+ ions-treated soil. The second subgroup consisted of 11 genera with low variation depending on the treatment. *Bacillus*, the most abundant genus, was clearly demonstrated to have the lowest frequency in the control and AgNPs-TA variants, while there was a visible increase after fungicide treatment and a very high increase after treatment with AgNPs-SBTC, AgNPs-SHSH, and Ag^+^ ions (Figure 4 and Figure 5).

There were moderate variations in the results of the analysis of bacterial community indicators (Table 2). The most deviant variants were fungicide (over 38,000) and Ag^+^ ions (less than 25,000 reads). The number of species decreased from 910 in the Ag^+^ ions to 769 in the fungicide-treated soil. The density of the community suggests that the variants with the most numerous dominants or containing distinct dominants are the fungicide (49), control (42), and AgNPs-SBTC (40) variants. The Ag^+^ ions variant had the lowest ratio (26). Based on the size of the ecological indicators used at the species level, slight changes were observed in all variants. The dominance rate was not more than 0.02, which indicates the absence of dominant species or, which is more justified in this type of research, a large number of singletons. Regarding the control, the Shannon diversity index and Pielou uniformity increased after the use of chemicals. The high Shannon uniformity factor, in particular, indicates the presence of a large number of species with a high uniformity factor at the same time. The Pielou uniformity index does indicate a high uniformity in species distribution, though about 25% inequality indicates that there is a group of taxa with a higher (e.g.,sub dominating) frequency, which applies to each variant. Despite this, these results indicate an increase in the number of low-frequency taxa and a decrease in the importance of dominant taxa the within chemically treated variants compared to the control.

#### 3.2.3. Biochemical Properties of Microbiota

**Figure 6:**
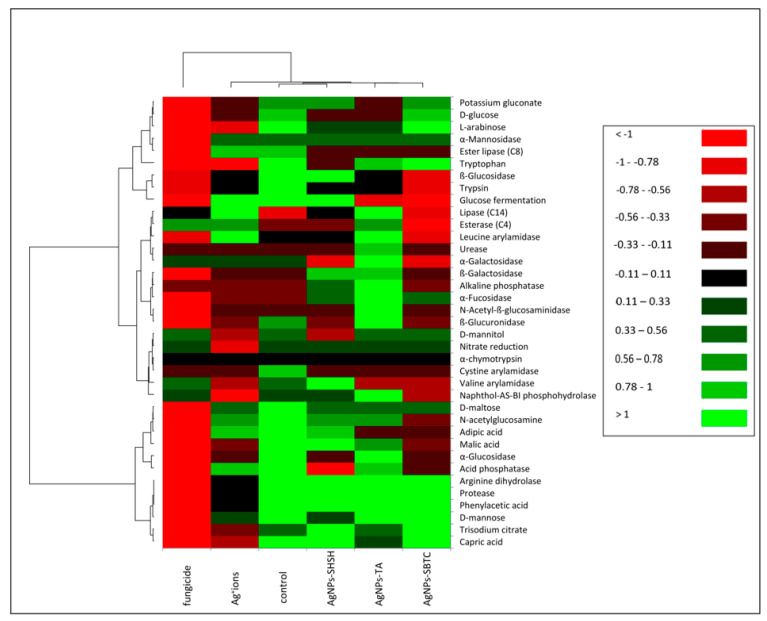
Heat map showing the potential of bacterial biochemical activity in the control and treated soils.

Soil enzymes are natural mediators and catalysts of soil processes, that is, the decomposition of organic matter, the formation and decomposition of humus, the release and making available of mineral substances to plants, the fixation of molecular nitrogen, as well as the flow of carbon, nitrogen, and other elements of the biochemical cycle. Enzymatic activity is considered to be the basic indicator of changes in the soil environment. The results obtained from the studies (Figure 6) indicate that AgNPs, Ag^+^ ions, and fungicides cause significant and differing changes in the biochemical potential of the treated soil. Treatment of the soil with AgNPs-SHSH resulted in the least significant changes in biochemical potential compared to the control. Significant changes were found in the AgNPs-TA and AgNPs-SBTC variants of the treatment. The biochemical potential of bacteria in the soil treated with Ag^+^ ions was clearly weaker and different from the control and the treatment with AgNPs. Fungicides suppressed the enzymatic activity of soil bacteria most strongly. After the application of AgNPs-SHSH, an increase in the activity of B-galactosidase, alkaline phosphatase, and a-fucosidase was observed, as well as a strong decrease in the activity of acid phosphatase and, to a lesser degree, that of a-glucosidase, cystine arylamidase, D-mannitol utilization, tryptophan metabolism, lipase ester (C8), B-glucuronidase, and D-glucose utilization. AgNPs-TA had an impact on an increase in the activity of chitinase, a-fucosidase, alkaline phosphatase, B-galactosidase, urease, leucine arylamidase, esterase (C4) and lipase (C14). In this treatment, there was a decrease in the activity of: adipic acid utilization, D-glucose and potassium gluconate, valine and cystine arylamidase, glucose fermentation, ester lipase (C8), and, to a lesser degree, B-glucosidase. In the treatment of soil with AgNPs-SBTC, no clear increase in any activity was observed compared to the control. There was a decrease in numerous activities, these being: B-glucosidase, glucose fermentation, cystine, leucine, and valine arylamidase, naphthol-AS-BI phosphohydrolase, a-glucosidase, acid phosphatase, chitinase, B-glucuronidase, B-galactosidase, lipase (C14), esterase (C4), glucose fermentation and utilization of malic acid, adipic acid and chitin. Treatment of the soil with Ag^+^ ions significantly decreased the biochemical potential of the microbiota, but in the case of the activity of lipase (C4), esterase (C14), and leucine arylamidase, an increase in activity compared to the control was observed. In the case of that treatment, acid phosphatase, ester lipase (C8), a-mannosidase and chitin utilization, D-mannose, D-maltose, adipic acid, and glucose fermentation did not undergo any significant change, and all remaining activities were significantly impaired.

In the soil treated with fungicide, the activity of the microbiota was nearly completely suppressed. Only the activity of esterase (C4) increased in relation to the control. In contrast, the activity of a-galactosidase, nitrate reductase, valine arylamidase, naphthol-AS-BI phosphohydrolase, and D-mannitol utilization remained at a similar level.

The results obtained indicate that Ag^+^ ions, and fungicide, in particular, significantly reduce the metabolic activity of the microbiota. At the same time, in the case of AgNPs, the changes observed may be considered to amount to a strong modification to metabolic processes in relation to untreated soil.

**Figure 7:**
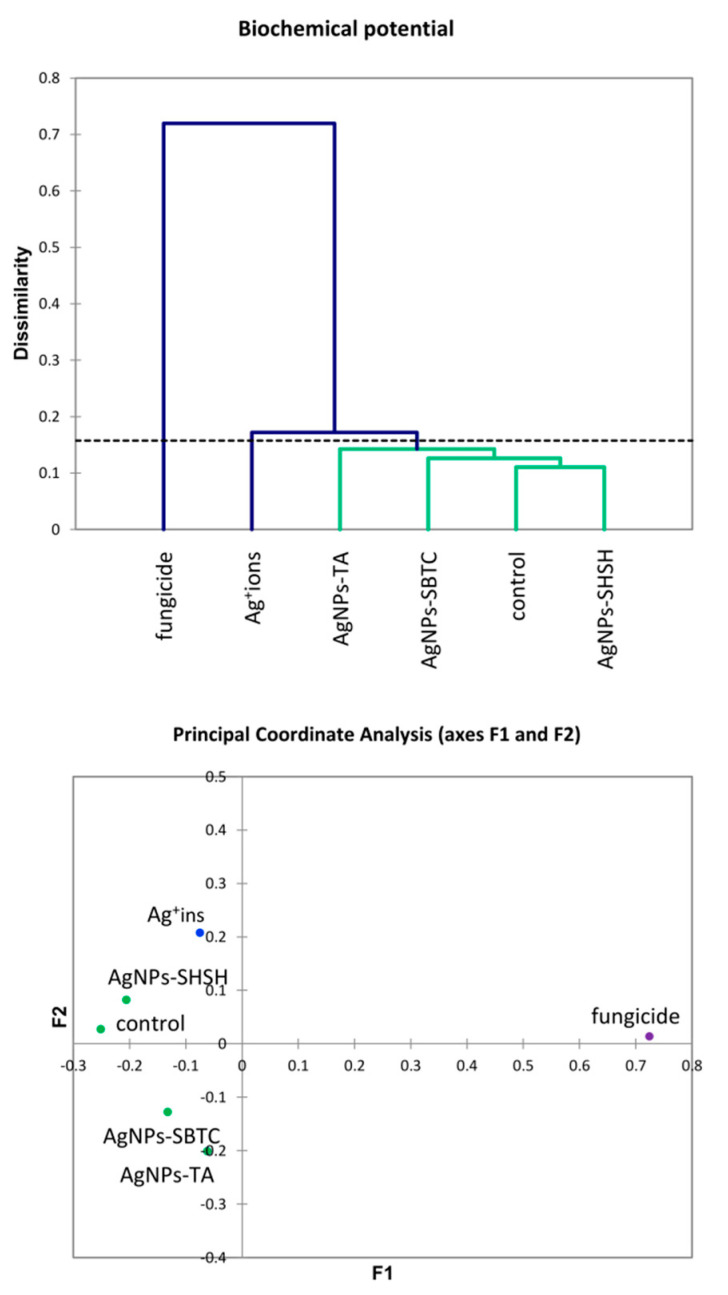
Agglomerative Hierarchical Clustering analysis (Bray and Curtis method) of soil bacteria biochemical activities in the control and treated soils (**top**) and the relationship between bacterial biochemical activities depending on the treatments according to PCoA (**bottom**).

The results of the AHC of biochemical properties (Figure 7) were partially similar to the PCA results for the microbiome. However, differences in the placement of the cut-off lines on the dendrograms result in a different grouping of variants. Generally, no clear association between the microbiome and biochemical parameters was observed in the case of the AgNPs-TA variant. At the level of genus, this variant had the microbiome with the most similar structure to the AgNPs-SHSH and fungicide variants. In contrast, biochemical parameters were similar to the AgNPs-SBTC variant, whose microbiome at the phylum level was different from all other variants. The results of PCoA (Figure 7) suggest some different observations compared to the results obtained with AHC. The value of determination of PCoA variables reached 91.2%, which points to the high reliability of the results obtained. In contrast to AHC, the PCoA results suggest that the control and the AgNPs-SHSH variant should be ungrouped from AgNPs-SBTC and AgNPs-TA variants. However, the distance from the center of the coordination axis is very small (<0.3 points).

**Figure 8:**
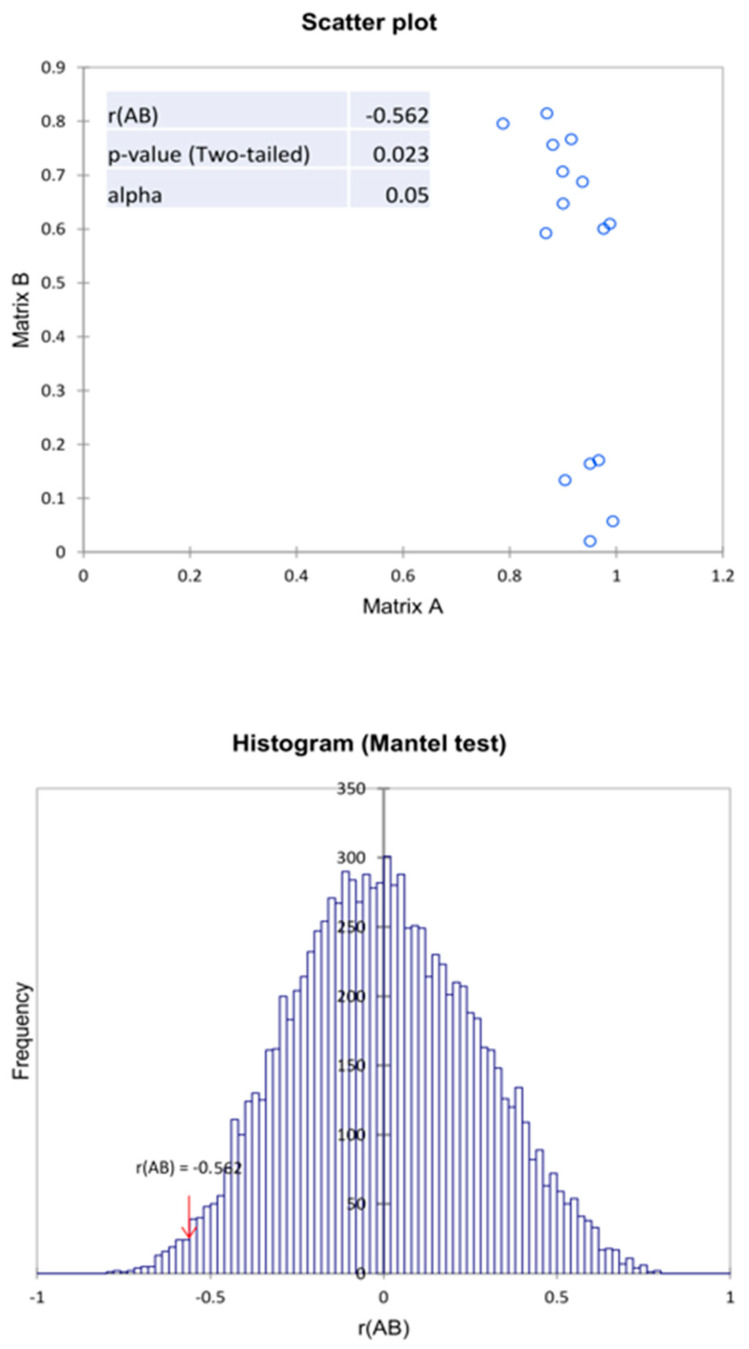
Mantel test of the correlation matrix of the bacteria with the biochemical activity in relation to the variants examined in the experiment.

To confirm the correlation between biochemical properties and the microbiome structure, the Mantel test was used. Based on the results obtained, a negative correlation value between two proximity matrices was observed, which is r(AB) = −0.566. This indicates a moderate negative correlation between the microbiome (genus) and the biochemical activity of these microbiomes in relation to the compounds tested. In addition, the value of *p* = 0.022 at α = 0.05, indicates the significance of the Mantel test regression coefficient at 10,000 permutations (Figure 8). Analyzing the above results, it can be concluded that when using agents that deplete the structure of the microbiome, the biochemical activity of these microbiomes increases. This is due to the strengthening of the dominant nature of only the most resistant types of microorganisms. This is due to the weakening of competition, antagonism, or the presence of spore microorganisms in the analyzed samples. It should be taken into account that the biochemical analysis method used is a method of describing the biochemical potential of a soil microbiome and not an assessment of existing biochemical (enzymatic) activity.

**Figure 9:**
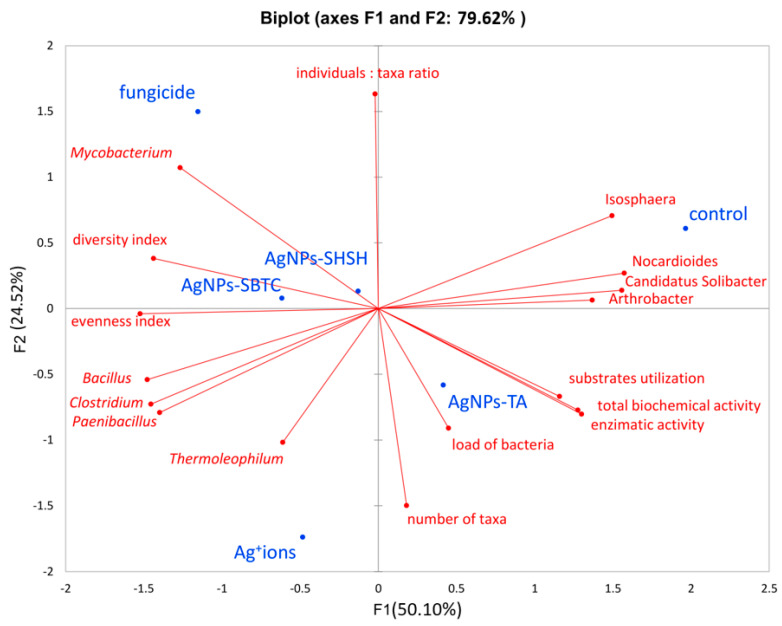
Principal Components Analysis based on alpha-diversity, biochemical and metataxonomic parameters.

Conducting an analysis of the main components explained 79.62% of the variability of results. Correct association (high values) with the respective variant was most precisely achieved for the control variant, AgNPs-TA, and fungicide. However, the proximity of the qualitative variable for the AgNPs-SHSH variant does not allow a relationship between individual qualitative variables and this variant to be observed. The only variants lying close to each other (and at the same time close to the center of the axis) were the AgNPs-SHSH and AgNPs-SBTC variants (about 0.1 relative to the F2 component and, on average, −0.3 relative to the F1 component), though the fungicide variant, while far away from them (more than 1 point relative to both axes) was in the same quarter of the chart. The other variants did not show strong similarity, and the AgNPs-TA variant was on the opposite side of both axes (positive F1 and negative F2), which indicated it to be opposite in nature to the above-mentioned three variants (especially fungicide). Only the control variant was in a positive relation to both axes (Figure 9).

Analyzing the relationship between variants and factors, it was found that the genera present in higher abundance, such as *Isosphera*, *Nocardioides*, *Candidatus* Solibacter, and *Arthrobacter,* are located on the positive axes F1 and F2 and are characteristic of the control object and, to a lesser extent, of the AgNPs-TA variant. High values of all biochemical activity parameters are also characteristic of these variants. In contrast, the location of the *Bacillus*, *Clostridium,* and *Paenibacillus* on the opposite side of the coordinate system points to a strong connection between the high frequency of these genera, especially with the Ag^+^ ions, as well as fungicide and AgNPs-SBTC variants (because F1 determines as much as 55.1% of variance). The above results also indicate a negative correlation between control-specific families and Ag^+^ ions. It was observed that the fungicide variant also had a higher abundance of *Mycobacterium* spp. and that the Ag^+^ ions variant had a higher abundance of *Thermoleophilum* spp.

The density of individuals was not clearly associated with any of the variants, while the high taxa number was characteristic of the AgNPs-TA and Ag^+^ ions variants. Higher values of ecological indicators expressing diversity and uniformity were characteristic for the AgNPs-SBTC, fungicide, and, to a lesser extent, the Ag+ ions and AgNPs-SHSH variants. It should also be noted that they were negatively correlated with types of characteristics of the control object. The total abundance of the genera *Nocardioides*, *Candididatus* Solibacter, and *Arthrobacter* are greater than *Bacillus*, *Clostridium,* and *Paenibacillus*, which indicates that the depletion of the participation of these three groups had an impact on the increase in the uniformity and diversity indicator (and thus a decrease in the level of dominance). One important observation is also the close connection between the high level of bacterial load with the AgNPs-TA variant, which is in agreement with the results obtained from the qPCR analysis (Figure 9). Based on a global observation in conjunction with the other statistical results, it can be concluded that the use of fungicides had the greatest impact on the bacterial microbiome and its potential activity. Next, the AgNPs-SBTC and AgNPs-SHSH, and Ag^+^ ions-treated variants had a significant impact, and the microbiome of the AgNP-TA-treated soil showed the lowest changes in relation to untreated soil, with there even being an improvement in enzymatic activity for some enzymes.

## 4. Discussion

### 4.1. Effect of AgNPs and Ag^+^ Ions on Soil Bacterial Community

The soil microbial community is responsible for a number of functions of soil ecosystems, such as the circulation of organic matter, soil structure, fertility, detoxification, support of plant growth, and both incidences and reduction in plant diseases. Bacteria carry out a lot of functional processes that are an integral part of maintaining environmental balance [50,51,52,53]. In this work, we have estimated a load of bacteria based on the number of copies of 16S rRNA genes. A proper analysis of the microbiome structure determination was carried out initially at the phylum level and in detail at the genera and species level and compared with the results of biochemical tests, which are often used in environmental studies. The results supported by multilevel statistical calculations allowed precise conclusions to be drawn regarding the positive and negative impacts of the treatments used.

In general, the destructive effects of mixtures of fungicides on the bacterial community and their biochemical activity in the soil and aquatic environments have already been confirmed [54,55]. However, the effects of Ag^+^ ions and AgNPs in a soil environment are diverse and not fully understood [29,56,57].

In the quantitative assessment, the results obtained show that only the fungicide resulted in a strong decrease in the number of bacteria compared to the control. Furthermore, in the qualitative assessment, the share of all types of persistent bacteria, i.e., *Bacillus*, *Paenibacillus* and *Clostridium,* clearly increased in all treated soils compared to the control and there was a visible decrease in the share of *Nocardioides*, *Arthrobacter,* and *Candidatus* Solibacter. Nevertheless, the most important observation in this work is the fact that any interference in the soil environment resulted in almost complete depletion of the microbiome in *Pseudomonas* bacteria, which are useful soil and rhizosphere microbiotics. Soni et al. [58] demonstrated that AgNPs are toxic to *Pseudomonas* bacteria in soil and induce oxidative and metabolic stress. Gupta et al. [59] have shown mycosynthesized AgNPs to have a strong antibacterial effect on *Pseudomonas putida*, a rhizospheric bacterium responsible for plant protection and bioremediation. In in vitro laboratory experiments with multidrug-resistant *Pseudomonas aeruginosa*, there has also been shown to be strong antibacterial and antibiofilm potential in concentrations from 1 µg·mL^−1^ of AgNPs produced by Hunan Anson Biotechnology Co., Ltd. after stabilization with cyclodextrin and Na_2_S [60], and produced using sodium citrate, ascorbate, and sodium borohydride [61]. Our studies confirm the high sensitivity of *Pseudomonas* to AgNPs independent of the type of their stabilizing agents.

Observations in our research have clearly differences between treatments on some other genera. The AgNPs-TA-treated soil showed the highest number of bacteria, which was associated with the growth of other non-sporulating types and the emergence of *Pedobacter*, *Bradyrhizobium*, and *Janibacter*. The use of fungicide resulted in an increase in the abundance of *Rhodanobacter*. It was surprising to observe the development of bacteria of the genus *Sphingobacterium* (actively producing extracellular polymeric substances—EPS surfactants) after soil treatment of AgNPs-SHSH and AgNPs-SBTC.

In various soil studies, the results for the microbiome structure also differ, making it difficult to draw clear conclusions [62,63].

### 4.2. Relations between AgNPs and the Microbiome

Searching for a relationship in the toxicity of AgNPs towards soil bacteria, several studies have assessed the soil factor to be decisive in this effect. Some ecotoxicological studies have shown a large reliable correlation between AgNPs toxicity and soil characteristics. Schlich and Hund-Rinke [64] showed that the toxicity of AgNPs is negatively correlated with an increase in pH, as well as clay content. In our work, we used sandy-clay, medium-acidic (pH 5.1) soil, which reflects the average impact of AgNPs on soil microorganisms.

Other studies assessed the doses of AgNPs used. In Samarajeewa et al. [62], a wide range of doses (60–2000 mg kg^−1^) of polyvinylpyrrolidone-coated (0.3%) AgNPs were tested on the soil microbiome. The strong negative impact on the soil microbiome was already confirmed at the lowest dose. Depending on the method, the lowest IC_50_ reached 19.9 mg·kg^−1,^ calculated as Ag. In this study, there was a clear change in ecological indicators after exposure to AgNPs. Unlike the results we obtained, the authors observed a decrease in microbiome diversity. A decrease in the abundance and enzymatic activity of soil bacteria in permanent agricultural pastureland at low concentrations of AgNPs has also been observed by McGee et al. [53], with an increase in the diversity of taxa similar to that seen in our studies. These observations concerned not only bacteria but also fungi and archaea in soil. Commercial AgNPs from SkySpring Nanomaterials Inc. (Houston, TX, USA) were used in the research. This confirms—as our own research—that the deposition of various AgNPs in the soil, even in small quantities, can permanently modify the community of microorganisms. This should be considered a universal conclusion independent of the type of AgNPs.

Shin et al. [65] showed that the presence of AgNPs significantly affects the enzymatic activity of soil. In their studies, in particular, urease and dehydrogenase activity, but also arylsulfatase and ß-glucosidase, were significantly associated with the presence of AgNPs. The first two enzymes were already inhibited at a concentration of 10 mg·kg^−1^ of dry soil. The effect of silver ions dissolved from AgNPs was not significant, indicating adverse effects caused by AgNPs alone. Their study suggests that AgNPs negatively affect soil exoenzyme activity, with urease activity being particularly sensitive to AgNPs. Significant dose-response (1, 10, and 100 mg·kg^−1^ soil) inhibitions of polyvinylpyrrolidone-coated AgNPs on enzyme activities have been observed by Yan et al. [66], with dehydrogenase more susceptible to treatment. This examination also confirmed changes in the bacterial and archaeal community structure. All studies conducted still confirm that AgNPs indeed have the potential to change the soil microbial community’s functional contributions and interfere with the ecological functions of soil.

The source of AgNPs in the soil may not only be engineered particles (produced and used in various technologies), but they may also occur naturally, e.g., due to transformed silver compounds occurring in sewage sludge. Research into the long-term effects of the deposition of this type of AgNPs in the soil has been conducted by Schlich et al. [67]. The exposure of plants and soil microbiome to different silver sulfide nanomaterials present in sewage sludges provided evidence of their potential to bring about change in the community of soil microorganisms and the bioavailability of these compounds to plants, their accumulation in the roots and aboveground parts of plants, which may result in AgNPs entering the food chain network. This is confirmed by studies by Kraas et al. [68] and Grün and Emmerling [69]. Whereby these types of AgNPs may exhibit toxic effects over the long term rather than the short term.

Zhai et al. [28] studied the influence of different shapes (plates, spheres, and rods) of AgNPs on the soil microbiome and biochemical features of the microbiome. The plate-type AgNPs were dispersions in polyvinylpyrrolidone. The nanosphere type was stabilized in polyoxyethylene glycerol trioleate and polyoxyethylene (20) sorbitan mono-laurate. The nanorod-type AgNPs used in the tests were in an aqueous solution containing polyvinylpyrrolidone, acrylic/acrylate copolymer, and polycarboxylate ether. The authors concluded that the main negative impact on the microbiome is due to the increase in concentration. However, the AgNPs shape is also important. Biochemical analysis of the bacterial community did not provide any conclusive results. Still, it was initially concluded that AgNPs inhibit the activity of enzymes responsible for the energy demand of bacterial cells, which was generally confirmed in this study for spherically shaped AgNPs because AgNPs-SHSH and AgNPs-SBTC impaired the action of enzymes responsible for the utilization of sugars.

A series of studies concerning various types of AgNPs have demonstrated the possibility of permanently modifying the bacterial microbiome of the treated soil. Still, knowledge resulting from this data remains disparate.

Independent of the type of AgNPs, the most important factor limiting the quantity and activity of bacteria should be the release of Ag^+^ ions. This was found to be the case in experiments conducted in vitro by Xiu et al. [70] for the model *Escherichia coli*. Numerous studies show that Ag^+^ ions can interact with sulfur-containing proteins in the bacterial cell wall, which may lead to compromised functionality. Moreover, the interaction of Ag^+^ ions with the thiol group of vital enzymes may result in their impaired function or even inactivation. Other mechanisms of toxicity are considered to be the adhesion of AgNPs to the bacteria’s surface altering the properties of the membrane and their penetration inside the cell, resulting in DNA damage [31,71]. Our test, regarding the aspect of the impact of Ag^+^ ions on modification of soil bacteria community, showed that, in complex communities (soil microbiome), the mere release of ions from AgNPs does not have to prove to be a highly toxic factor, yet remains a factor that significantly modifies the composition of the bacterial community. In addition to this, we found that Ag^+^ ions have an effect on an increase in the occurrence and activity of numerous bacteria, the presence of which in untreated soil was inhibited by dominant genera of bacteria. The treatment of soil with Ag^+^ ions and AgNPs resulted in a significant qualitative change and an increase in the abundance of certain bacteria, which were not present or occurred in very low intensity in the control soil.

### 4.3. Effect of Chemical Structure of Stabilizing Layers of Negatively Charged AgNPs on Soil Bacteria and the Enzymatic Activity of Bacteria

The premise of our study was to check how negatively charged AgNPs, which are similar in terms of physico-chemical characteristics but different in terms of the chemical structure of stabilizing layers, modify the assessed soil bacterial community. Our studies show that the chemical structure of stabilizing layers of AgNPs differentiates them in terms of the way they have an effect on soil bacteria and the enzymatic activity of bacteria. The quantitative and qualitative differences found in this case should result not only from the level of ion release but also from the chemical structure of the stabilizing layers. The tannic acid (TA), sodium borohydride (SB) in the presence of trisodium citrate (TC), and sodium hypophosphite (SH) in the presence of sodium hexametaphosphate used for preparation in our studies have an effect on the type of chemical structure of stabilizing layers of AgNPs, and that had a differentiating effect in terms of impact on the soil microbiome.

TA is a compound that has the capacity to bind to proteins and may give rise to a process of denaturation of bacterial protein. TA also has a strong iron binding capacity, meaning it is able to inhibit the growth of bacteria needing that element under in vitro conditions, as was proven by Chung et al. [72]. Examining soil with an iron-deficient rhizosphere, Yang and Crowley [73] found there to be a significant response on the part of the bacteria community and changes dependent on phytosiderophores secreted by plants and the studied root zone. In the study, the direction of the quantitative and qualitative changes caused by iron deficiency was not, however determined. In our studies, AgNPs synthesized with TA resulted in a very significant quantitative increase in bacteria compared to the control and a qualitatively significant increase in Proteobacteria compared to all the other treatments. In addition to many pathogenic species, Proteobacteria include many bacteria that are responsible for nitrogen fixation. This allows us to conclude that AgNPs with stabilizing layers containing TA is not toxic to the soil bacteria community.

For the production of AgNPs-SBTC, SB and TC were used, which differ significantly in terms of their effect on bacteria. SB is a highly chemoselective reducing agent. It has been found to be a case that, as one of the reducing agents, SB can weaken the degradation of DNA caused, for example, by antibiotics [74,75]. Interaction with DNA is mentioned as one of the biocidal factors in the action of different types of AgNPs. The other reducing agent used in the production of AgNPs-SBTC, namely TC from the citrates group, can form chemical compounds with metal ions, is a strong chelating agent, and has alkalizing properties. A series of tests have confirmed the antibacterial activity of sodium citrates. It has also been found to be the case that certain metal ions, namely Mg^2+^ and Ca^2+^, the presence of which enhance antibacterial properties, have an effect on the activity of TC. In contrast, this activity is not affected by a pH in the range of 5.0 to 8.0, which is an exception and goes against the popular belief that undissociated acid can be an effective antiseptic. This suggests that the mechanism of bacterial inhibition differs in the case of TC from the mechanism in acids and is independent of pH. There are considered to be two different mechanisms to that activity: direct destruction of the bacterial outer membrane (cationic bonding of the molecule to components of the cell wall, which disrupts the function of the membrane and ultimately leads to leakage of cytoplasmic components) or that TC captures Ca^2+^ and Mg^2+^ ions that are necessary for bacterial growth (capturing the necessary nutrients may be associated with bacteriostasis). In general, however, this activity is attributed to chelating properties [76,77]. In soil treated with AgNPs-SBTC, we found there to be a significant increase in the proportion of Bacteroidetes and Firmicutes. These two groups include anaerobic, often toxicogenic bacteria, many of which are resistant to adverse environmental conditions and form endospores. A significant increase in this type of bacteria and a decrease in the proportion of bacteria more sensitive to pollutants in the soil may be evidence of the toxic effect of AgNPs-SBTC related to the release of ions in synergy with the action of TC.

The AgNPs-SHSH were produced using hexametaphosphate and sodium hypophosphite. These compounds at high in vitro concentrations have been assessed to inhibit the growth in particular of gram-positive pathogenic bacteria and, to a lesser extent, that of gram-negative bacteria [78,79]. Analyzing the results of our studies, we found that, compared to the control, AgNPs-SHSH caused a significant decrease in the proportion of 11 of the designated genera of gram-positive bacteria and an increase in six of them. In the case of gram-negative bacteria, there was a decrease in five genera and an increase in seven of them.

Some responses to the soil treatment used in our research at the species level allowed for a more in-depth interpretation of the results (Table S1). Changes found in the occurrence and abundance of certain species were due to treatment with all forms of silver or all types of AgNPs. Modifications were also found for the treatment with Ag^+^ ions only, as well as changes due to only one type or some types of AgNPs.

The strongest negative effect of all forms of silver applied was observed in relation to *Arthrobacter* sp. FB24 i.e., from >4% in the control to <0.02% under the effect of each Ag treatment. The fungicide reduced this species to ~1%. This was the only representative of *Arthrobacter* spp., the proportion of which underwent such a significant decrease. This strain is characteristic of environments rich in petroleum hydrocarbons and extreme metal contaminations. It has been found to be distinct from other species of *Arthrobacter* both genetically and in terms of detoxification [80,81,82,83,84]. Despite a highly efficient detoxification mechanism for metals and organic compounds, a marked reduction in the abundance of this species was a surprise, and further research is needed in this area. Nevertheless, this result indicates that it is not the type of AgNP stabilizer but the mere presence of Ag^+^ ions that is toxic for this strain. It should also be emphasized that a comparison of the metabolism [85] of *Arthrobacter* sp. FB24 with other species present in our samples showed no major differences. However, this strain did lack some genes (ATP-binding cassette) responsible for intracellular detoxification and the excretion of toxic substances.

Among the other remaining species, a clear reduction due to each of the agents applied was observed in the case of *Pseudomonas* spp., *Nocardioides* sp. MTD22, *Frankia* sp. and *Candidatus* Solibacter usitatus. The presence of *Terrabacter tumescens* was particularly reduced by fungicide and AgNPs. Despite the fact that we were not able to find genomic data in KEGG (Kyoto Encyclopedia of Genes and Genomes) for all species identified by us, it should be noted that these taxa contribute significantly to the nitrogen cycle in the environment. *Nocardioides* spp. has a complete ammonification pathway and, at the same time, has a detoxification capacity for some metals [86], while *Frankia* spp. is a microorganism that plays a significant role in nitrogen-fixing in the atmosphere (N-fixation) [87]. Metal homeostasis plays a key role in the success of symbiosis and assimilation in obtaining the trace metals necessary for these processes. Chelating molecules (metallophores) are produced and secreted by microorganisms in order to efficiently bind a given metal ion. Bacteria are also able to release many organic ligands derived from dissolved organic matter with a broad spectra of affinities with necessary or toxic metals. Based on the results of our research, it can be assumed that AgNPs are capable of disrupting these processes and, above all, the basic function of bacteria in ecosystems related to nitrogen fixation. However, this does not seem to be dependent on the chemical structure of stabilizing layers of different types of AgNPs, but rather on the release of Ag^+^ ions. The reduction in the population of bacteria responsible for the transformation of nitrogen in the soil by AgNPs related to the release of Ag^+^ ions has also been confirmed in studies by Juan et al. [88].

However, in our test, not all species with high detoxification potential were limited by soil treatment with AgNPs. In the case of *Rhodanobacter lindaniclasticus*, which has been confirmed to have the potential to degrade and metabolize pesticides that are harmful to the environment [89], and the tolerance of which to AgNPs has moreover also been confirmed [90], an increase in population size was observed in the variant where fungicide (>1.3%) and AgNPs (~0.7%) were applied. In the control soil and in the variant with treatment with Ag^+^ ions, the population size was <0.01%. Due to the small amount of genomic data on *Rhodanobacter lindaniclasticus* other related species were also used to analyze the metabolic pathways (KEGG). It was observed that bacteria of this genus have extensive apparatus for the detoxification and utilization of organic compounds, which may explain the increase in population size in the case of fungicides and AgNPs. It is also worth drawing attention to the fact that bacteria of this genus are denitrifiers. A significant increase in the population of *Rhodanobacter* spp. was also observed by McGee et al. [53] when treating the soil with relatively low concentrations (1–50 mg kg^−1^) of AgNPs (spherical, 20–30 nm size) obtained from Sky Spring Nanomaterials Inc.

The conclusion is that AgNPs present in the soil can reduce the populations of some metal-detoxifying bacteria and stimulate some species capable of detoxifying organic pollutants such as pesticides. A similar impact stimulating population development to that with *R. lindaniclasticus* was observed with *Sphingobacteriaceae bacterium* SOC A20(36), considered to be bacteria capable of degrading high molecular weight carbon compounds.

Despite the fact that, in the majority of cases, the AgNPs used in the study were shown to have an effect on the soil microbiome independently of the chemical structure of stabilizing layers, a number of clear associations were observed between species population size and the type of AgNPs. In most cases, this was a stimulating effect. Ag^+^ ions increased the proportion of *Thermoleophilum album*, *Bacillus circulans*, *Clostridium botulinum*, *Arthrobacter globiformis*, *Chthoniobacter flavus*, *Geodermatophilus obscurus,* and *Paenibacillus alginolyticus*. In the case of AgNPs, the following relationships were noted: AgNPs-TA increased the proportion of *A. globiformis* and *Pedobacterheparinus*;. In contrast, AgNPs-SBTC increased that of *A. globiformis*, *Bacillus megaterium,* and *Sphingobacterium* sp. TN19; and AgNPs-SHSH increased that of *Sphingobacterium* sp. TN19. However, a decrease in proportion due to only one type of NP was observed for *Atopobium minutum* due to AgNPs-TA.

An increase in *A. globiformis* abundance in the AgNPs- and Ag^+^ ions-treated soils indicatethe potential of Fe immobilization and disruption of Fe ion uptake by microorganisms. *A. globiformis* is a habitat-specific species with limited iron content, secreting efficient iron-chelating molecules with a high affinity for dissolving and mobilizing that element. It is a species tipped to be a promising bioinoculant in reducing iron stress in crops [91].

*Sphingobacterium* sp. TN19 stimulated by AgNPs-SBTC and AgNPs-SHSH is a sphingosine-containing species characterized by a high concentration of sphingophospholipids as lipid components. Sphingolipids perform different functions at many levels of the organization of organisms: namely at anatomical, histological, molecular, and cytological levels. Playing a part in the composition of cell membranes as elements of lipid rafts, they can be responsible for the penetration of viruses into cells. Many studies show that sphingolipids may be conducive to the survival and replication of bacteria under stress conditions, especially with regard to increasing the integrity of the cell membrane [92].

Summarizing the results of the studies conducted with a view to qualitative assessment, different types of ANPs reduce bacterial populations in the soil microbiome in a similar manner but may stimulate the development of different taxa, and this is, among other things, dependent on the chemical structure of stabilizing layers. We confirmed that similarly to under in vitro conditions [70], the level of ion release is responsible for the bacteriostatic action of AgNPs and quantitative changes in the bacterial community in the soil, and the level of ion release is determined, amongst other things, by the chemical structure of the stabilizing layers. In addition to this, we found that the chemical structure of the stabilizing layers may modify specific metabolic paths of bacteria and be decisive in bringing about a qualitative change in the soil bacterial community. It is related to the drift in the entire soil microbiome, which can significantly change the soil biochemistry, and thus affect plants and the circulation of biogenic elements in the soil environment. These changes in population size ought to be examined, and detailed chemical, enzymatic and metatranscriptomic studies of the soil environment treated with AgNPs should be carried out, taking into account the chemical structure of stabilizing layers in order to predict the long-term effects of such changes. It may also be worth underlining that changes resulting from the treatment of soil with low concentrations of AgNPs may stimulate populations of some species of bacteria useful in the bioremediation of soil.

## 5. Conclusions

The studies conducted showed a different mechanism of action of the tested pesticide and of silver compounds on the soil microbiome. The application of fungicide had the strongest negative impact on the bacterial community and its biochemical activity. AgNPs-SBTC and AgNPs-SHSH and Ag^+^ ions also had a significant effect. The soil microbiome treated with AgNPs-TA showed the smallest changes compared to untreated soil and even a more numerous bacterial communities and better enzymatic activity. There was a clearly greater proportion of all types of persistent bacteria, i.e., *Bacillus*, *Paenibacillus* and *Clostridium*, in all the treated soils compared to the control. At the same time, a visible decrease was seen in the share of *Nocardioides*, *Arthrobacter,* and *Candidatus* Solibacter. One important observation is that all the compounds used in the research resulted in a complete depletion of the microbiome in *Pseudomonas*, which are fundamental soil microbiotics. Ag^+^ ions, and especially the fungicide, significantly decreased the metabolic activity of the microbiota, where, in the case of AgNPs, the observed changes can be described as a strong modification of metabolic processes. An analysis of indices of dominance, diversity, and evenness of bacterial communities showed there to be an increase in the number of low-frequency taxa and a decrease in the importance of dominant taxa in the chemically treated variants compared to the control. It was found that, in complex communities (soil microbiome), the mere release of Ag^+^ ions from AgNPs does not have to prove to be a highly toxic factor but remains a factor that significantly modifies the composition of the bacterial community. In addition to this, the release of Ag+ ions have an effect on an increase in the occurrence and activity of numerous bacteria, the presence of which in untreated soil was inhibited by dominant genera of bacteria.

Based on the results of our research, it can be assumed that AgNPs, by having an effect on the homeostasis of metals in the soil, are able to disturb processes of nitrogen assimilation by bacteria and the release by them of chelating compounds and ligands derived from dissolved organic matter with a wide range of affinities for essential or toxic metals. However, this does not seem to be directly dependent on chemically different stabilizers adsorbed on the surface of AgNPs, but rather on the level of release of Ag^+^ ions.

The research results obtained confirm that similarly to under in vitro conditions, it is above all, the level of ion release that is responsible for the bacteriostatic action of AgNPs and quantitative changes in the bacterial community in the soil, and the level of ion release is in turn determined by the chemical structure of the stabilizing layers. In addition to this, the results of our research indicate that differences in the chemical structure of the stabilizing layers of AgNPs may modify certain specific metabolic paths of bacteria and be decisive in bringing about a qualitative change in the soil bacterial community, which is no longer related to the release of Ag^+^ ions.

## Figures and Tables

**Table 1:** Selected physicochemical properties of the AgNPs and their aqueous suspensions.

Parameter of Stock Suspension ^†^/AgNPs ^‡^	Type		
	AgNPs-TA	AgNPs-SBTC	AgNPs-SHSH
^†^ concentration [mg L^−1^]	100	100	100
^†^ pH	5.8	6.3	6.3
^†^ plasmon absorption maximum [nm]	414	412	404
^‡^ diameter determined from TEM [nm]	16 ± 4	15 ± 3	14 ± 2
^‡^ diffusion coefficient [μm^2^s^−1^] ^§^	30.7 ± 0.3	31.1 ± 0.3	37.7 ± 0.5
^‡^ hydrodynamic diameter [nm] ^§^	16 ± 3	16 ± 3	13 ± 4
^‡^ polydispersity index (PdI) ^§^	0.25	0.19	0.31
^‡^ electrophoretic mobility [μmcm (Vs)^−1^] ^§^	−3.97 ± 0.40	−3.89 ± 0.16	−3.39 ± 0.09
^‡^ zeta potential [mV] ^§^	−77 ± 4	−75 ± 2	−63 ± 1

^§^ determined at pH 5.8–6.3 and a temperature of 25 °C (ionic strength was not regulated by the addition of any additional electrolytes; therefore, based on the conductivity measurements, it was assumed that it was equal ca. 10^−5^ M). ^†^ and ^‡^ means characteristics concerning of stock suspension (†) and characteristics concerning of AgNPs (‡).

**Table 2:** Community indices for bacterial operational taxonomic units in the control and treated soils.

Indicator	Control	Treatment				
		Fungicide	Ag^+^ Ions	AgNPs-TA	AgNPs-SBTC	AgNPs-SHSH
Number of readings after filtration	34,256	38,124	24,148	31,796	30,879	30,605
Number of species	808	769	910	845	771	829
Density index	42.4	49.6	26.5	37.6	40.1	36.9
Simpson’s dominance index	0.02	0.01	0.02	0.01	0.01	0.01
Shannon’s diversity index	4.83	5.31	5.05	5.17	5.32	5.23
Pielou’s evenness index	0.71	0.78	0.76	0.77	0.78	0.77

## Data Availability

Data supporting reported results can be obtained from the authors on request.

## Supplementary Materials

The following supporting information can be downloaded at: https://www.mdpi.com/article/10.3390/ijerph192114438/s1, Figure S1: Proportion analysis (soil untreated vs. treated) of metagenome on genus level; Table S1: Shifts in soil bacteria structure depending on the treatment on species level.
