# Supplementary material for: Chemical Structure of Stabilizing Layers of Negatively Charged Silver Nanoparticles as an Effector of Shifts in Soil Bacterial Microbiome under Short-Term Exposure
Source: Int J Environ Res Public Health. 2022 Nov 4;19(21):14438. doi: 10.3390/ijerph192114438 (PMC9658158; doi:10.3390/ijerph192114438)
Supplement: Supplementary file 1 [file ijerph-19-14438-s001.zip › ijerph-1979134-supplementary.pdf]

Figure S1. Proportion analysis (soil untreated vs. treated) of metagenome on genus level.

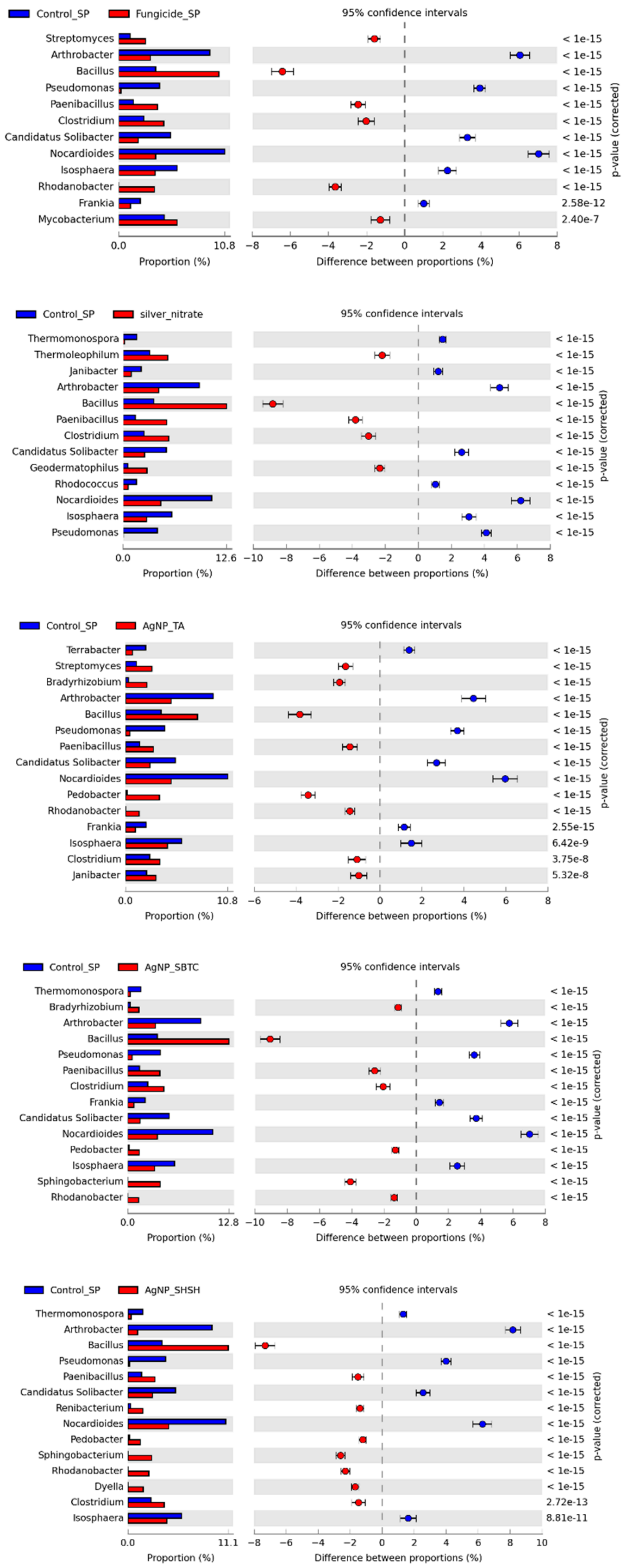

Table S1. Shifts in soil bacteria structure depending on the treatment on species level.

Treatment							SPECIES
Control	Fungicide	Silver nitrate	AgNP-TA	AgNP-SBTC	AgNP-SHSH	MEAN	
29.49	37.73	27.42	34.05	37.53	46.65	35.72	uncultured bacterium
0.92	11.31	0.69	16.17	2.93	2.89	6.18	uncultured gamma proteobacterium
4.85	7.17	0.65	3.67	3.62	1.89	3.91	uncultured soil bacterium
3.30	1.38	1.76	1.75	1.62	1.82	1.95	Isosphaera pallida
1.80	1.54	3.35	1.58	1.58	1.27	1.79	Thermoleophilum album
2.08	0.90	2.94	1.72	2.02	1.11	1.73	uncultured delta proteobacterium
0.81	1.45	3.22	1.30	1.21	1.56	1.51	Bacillus circulans
2.93	0.74	1.63	1.02	0.74	1.14	1.36	Candidatus Solibacter usitatus
1.02	1.20	1.86	1.36	1.39	1.01	1.28	uncultured alpha proteobacterium
1.77	1.10	0.53	0.81	1.35	1.00	1.13	uncultured beta proteobacterium
3.07	0.31	0.93	0.61	0.39	0.53	0.99	Nocardioides sp. MTD22
1.23	0.51	0.62	1.27	0.97	1.28	0.98	Janibacter sp. BY48
1.30	0.84	1.14	0.92	0.80	0.82	0.97	Candidatus Koribacter versatilis
1.77	0.58	1.06	0.77	0.73	0.75	0.94	Nocardioides albus
4.00	0.84	0.01	0.01	0.01	0.02	0.90	Arthrobacter sp. FB24
0.76	0.69	1.52	0.60	0.80	0.87	0.84	Clostridium botulinum
0.19	0.06	2.30	1.42	1.21	0.09	0.79	Arthrobacter globiformis
0.78	0.43	1.40	0.46	0.51	0.52	0.65	Chthoniobacter flavus
1.22	0.42	0.80	0.41	0.36	0.67	0.64	Frankia sp.
0.00	1.37	0.00	0.57	0.65	0.97	0.63	Rhodanobacter lindaniclasticus
1.71	0.32	0.60	0.19	0.15	0.39	0.57	uncultured proteobacterium
0.32	0.32	1.80	0.54	0.19	0.53	0.56	Geodermatophilus obscurus
0.26	0.61	0.94	0.26	0.52	0.58	0.51	uncultured Rhodocyclaceae bacterium
0.02	0.53	0.07	0.71	0.85	0.71	0.49	Sphingobacteriaceae bacterium SOC A20(36)
0.11	0.32	0.24	0.23	1.60	0.47	0.49	Bacillus megaterium
1.02	0.28	0.90	0.16	0.34	0.27	0.48	Terrabacter tumescens

0.54	0.46	0.75	0.38	0.41	0.28	0.46	Seinonella peptonophila
0.04	0.19	0.02	1.38	0.54	0.55	0.46	Pedobacter heparinus
0.78	0.23	0.95	0.33	0.26	0.28	0.45	bacterium Ellin514
0.00	0.06	0.05	0.06	1.54	1.02	0.45	Sphingobacterium sp. TN19
0.19	0.36	1.02	0.30	0.54	0.26	0.42	Paenibacillus alginolyticus
0.41	0.25	0.86	0.32	0.33	0.34	0.40	Pirellula staleyi
0.41	0.45	0.58	0.15	0.42	0.31	0.38	Atopobium minutum
0.93	0.50	0.12	0.30	0.15	0.15	0.38	Thermomonospora curvata
0.55	0.49	0.24	0.33	0.27	0.29	0.37	uncultured forest soil bacterium
0.59	0.19	0.60	0.31	0.25	0.36	0.37	Pseudonocardia zijingensis
0.36	0.35	0.41	0.43	0.34	0.10	0.33	Ruminococcus bromii
0.17	0.43	0.52	0.21	0.35	0.32	0.33	Micromonospora chokoriensis
0.22	0.18	0.38	0.39	0.45	0.40	0.33	Streptosporangium roseum
0.17	0.30	0.46	0.24	0.43	0.28	0.31	Paenibacillus polymyxa
1.66	0.00	0.00	0.00	0.00	0.04	0.31	Pseudomonas sp. HS100
0.27	0.24	0.30	0.25	0.33	0.29	0.28	Sporanaerobacter acetigenes
0.20	0.15	0.69	0.16	0.25	0.25	0.26	Microbacterium aurum
0.06	0.32	0.71	0.06	0.40	0.08	0.25	Bacillus sp. SH3
0.24	0.24	0.30	0.27	0.23	0.22	0.25	Nocardiopsis alba
0.15	0.14	0.05	0.13	0.78	0.22	0.25	Terrabacter sp. YK3
0.44	0.10	0.15	0.05	0.06	0.67	0.24	Bacillus flexus
0.25	0.23	0.22	0.26	0.19	0.31	0.24	Roseiflexus castenholzii
0.14	0.21	0.46	0.21	0.27	0.22	0.24	Bacillus simplex
0.38	0.30	0.34	0.10	0.16	0.12	0.24	Nocardioides sp.
0.25	0.12	0.16	0.15	0.56	0.17	0.23	Rhodococcus opacus
0.32	0.04	0.36	0.23	0.26	0.25	0.23	Nocardioides sp. DN36
0.07	0.24	0.36	0.19	0.35	0.23	0.23	Ureibacillus thermosphaericus
0.20	0.15	0.35	0.29	0.23	0.19	0.23	Desulfitobacterium hafniense
0.17	0.23	0.55	0.22	0.12	0.13	0.23	Desulfonispora thiosulfatigenes
0.16	0.13	0.35	0.33	0.10	0.32	0.22	Microlunatus phosphovorus

0.14	0.31	0.10	0.22	0.28	0.21	0.22	Bacillus cereus
0.20	0.17	0.21	0.29	0.22	0.18	0.21	Rubrobacter xylanophilus
0.43	0.12	0.10	0.17	0.21	0.17	0.20	Arthrobacter oxydans
0.06	0.19	0.63	0.06	0.33	0.06	0.20	Kribbella jejuensis
